# Toxicity Profiles and Survival Outcomes Among Patients With Nonmetastatic Nasopharyngeal Carcinoma Treated With Intensity-Modulated Proton Therapy vs Intensity-Modulated Radiation Therapy

**DOI:** 10.1001/jamanetworkopen.2021.13205

**Published:** 2021-06-18

**Authors:** Xingzhe Li, Sarin Kitpanit, Anna Lee, Dennis Mah, Kevin Sine, Eric J. Sherman, Lara A. Dunn, Loren S. Michel, James Fetten, Kaveh Zakeri, Yao Yu, Linda Chen, Jung Julie Kang, Daphna Y. Gelblum, Sean M. McBride, Chiaojung J. Tsai, Nadeem Riaz, Nancy Y. Lee

**Affiliations:** 1 Department of Radiation Oncology, Memorial Sloan Kettering Cancer Center, New York, New York; 2 Division of Radiation Oncology, Department of Radiology, Faculty of Medicine, Chulalongkorn University, King Chulalongkorn Memorial Hospital, Thai Red Cross Society, Bangkok, Thailand; 3 Department of Radiation Oncology, Division of Radiation Oncology, MD Anderson Cancer Center, Houston, Texas; 4 Department of Medicine, Memorial Sloan Kettering Cancer Center, New York, New York; 5 ProCure Proton Therapy Center, Somerset, NJ

## Abstract

**Importance:**

Patients with nonmetastatic nasopharyngeal carcinoma (NPC) are primarily treated by radiotherapy with curative intent with or without chemotherapy and often experience substantial treatment-related toxic effects even with modern radiation techniques, such as intensity-modulated radiation therapy (IMRT). Intensity-modulated proton therapy (IMPT) may improve the toxicity profile; however, there is a paucity of data given the limited availability of IMPT in regions with endemic NPC.

**Objective:**

To compare toxic effects and oncologic outcomes among patients with newly diagnosed nonmetastatic NPC when treated with IMPT vs IMRT with or without chemotherapy.

**Design, Setting, and Participants:**

This retrospective cohort study included 77 patients with newly diagnosed nonmetastatic NPC who received curative-intent radiotherapy with IMPT or IMRT at a tertiary academic cancer center from January 1, 2016, to December 31, 2019. Forty-eight patients with Epstein-Barr virus (EBV)–positive tumors were included in a 1:1 propensity score–matched analysis for survival outcomes. The end of the follow-up period was March 31, 2021.

**Exposures:**

IMPT vs IMRT with or without chemotherapy.

**Main Outcomes and Measures:**

The main outcomes were the incidence of acute and chronic treatment-related adverse events (AEs) and oncologic outcomes, including locoregional failure-free survival (LRFS), progression-free survival (PFS), and overall survival (OS).

**Results:**

We identified 77 patients (25 [32.5%] women; 52 [67.5%] men; median [interquartile range] age, 48.7 [42.2-60.3] years), among whom 28 (36.4%) were treated with IMPT and 49 (63.6%) were treated with IMRT. Median (interquartile range) follow-up was 30.3 (17.9-41.5) months. On multivariable logistic regression analyses, IMPT was associated with lower likelihood of developing grade 2 or higher acute AEs compared with IMRT (odds ratio [OR], 0.15; 95% CI, 0.03-0.60; *P* = .01). Only 1 case (3.8%) of a chronic grade 3 or higher AE occurred in the IMPT group compared with 8 cases (16.3%) in the IMRT group (OR, 0.21; 95% CI, 0.01-1.21; *P* = .15). Propensity score matching generated a balanced cohort of 48 patients (24 IMPT vs 24 IMRT) and found similar PFS in the IMPT and IMRT groups (2-year PFS, 95.7% [95% CI, 87.7%-100%] vs 76.7% [95% CI, 60.7%-97.0%]; hazard ratio [HR], 0.31; 95% CI, 0.07-1.47; *P* = .14). No locoregional recurrence or death was observed in the IMPT group from the matched cohort. Two-year LRFS was 100% (95% CI, 100%-100%) in the IMPT group and 86.2% (95% CI, 72.8%-100%) in the IMRT group (*P* = .08). Three-year OS was 100% (95% CI, 100%-100%) in the IMPT group and 94.1% (95% CI, 83.6%-100%) in the IMRT group (*P* = .42). Smoking history was the only clinical factor significantly associated with both poor LRFS (HR, 63.37; 95% CI, 3.25-1236.13; *P* = .006) and poor PFS (HR, 6.33; 95% CI, 1.16-34.57; *P* = .03) on multivariable analyses.

**Conclusions and Relevance:**

In this study, curative-intent radiotherapy with IMPT for nonmetastatic NPC was associated with significantly reduced acute toxicity burden in comparison with IMRT, with rare late complications and excellent oncologic outcomes, including 100% locoregional control at 2 years. Prospective trials are warranted to direct the optimal patient selection for IMPT as the primary radiotherapy modality for nonmetastatic NPC.

## Key Points

**Question:**

Is intensity-modulated proton therapy (IMPT) associated with fewer treatment-related adverse events and comparable oncologic outcomes for patients with nonmetastatic nasopharyngeal carcinoma (NPC) compared with patients treated with intensity-modulated radiation therapy (IMRT)?

**Findings:**

In this cohort study of 77 patients with nonmetastatic NPC treated with curative-intent radiotherapy, IMPT treatment was associated with significantly fewer acute adverse events compared with standard-of-care IMRT, with rare late complications. Propensity score–matched analysis demonstrated equally excellent oncologic outcomes in both groups, including 100% locoregional control rate at 2 years in the IMPT group.

**Meaning:**

These findings suggest that IMPT should be discussed with patients as the potential primary radiotherapy modality for nonmetastatic NPC when it is available because it was associated with less acute toxicity burden compared with IMRT, with potential oncologic benefit in locoregional control.

## Teaser

This cohort study compares toxic effects and oncologic outcomes among patients with newly diagnosed nonmetastatic nasopharyngeal carcinoma (NPC) when treated with intensity-modulated proton therapy (IMPT) vs intensity-modulated radiation therapy (IMRT) with or without chemotherapy.

## Introduction

Nasopharyngeal carcinoma (NPC) is a distinctive type of head and neck cancer endemic to East and Southeast Asia likely due to genetic and environmental predispositions.^1^ Radiotherapy and chemotherapy have been well established as the fundamental pillars of multidisciplinary treatment for nonmetastatic NPC, significantly improving the survival outcomes of patients with NPC during the past 2 decades.^2,3^ Advanced radiotherapy (RT) techniques, such as intensity-modulated RT (IMRT), have been shown to decrease treatment-related toxic effects for patients with NPC.^4^ However, 50% to 75% of patients with NPC treated with chemoradiotherapy using IMRT experienced acute grade 3 or 4 adverse events (AEs), and 10% to 20% of surviving patients may experience serious late complications, such as feeding tube dependency and tissue necrosis.^4,5^ Today, patients with NPC have longer survival as a result of improvements in both primary treatment regimens and systemic options in the recurrent and metastatic setting.^6,7,8^ Therefore, the toxicity burden from primary treatment, such as chemoradiotherapy, has significant quality-of-life implications.^9^

Proton beam RT delivers minimal dose to normal tissues behind the tumor after depositing most of its energy in the target, whereas conventional photon RT still delivers a moderate amount of radiation to normal tissues along its path. In the treatment of head and neck cancer, this unique feature of proton beam RT is distinctively advantageous because the tumor is usually close to normal tissues with complex anatomy.^10,11^ Intensity-modulated proton therapy (IMPT) is the most advanced form of proton beam RT. It uses multiple proton beams from different angles with intensity modulation and optimization to achieve a high degree of conformality, therefore being able to deliver a high dose to the tumor target while protecting normal tissues in the vicinity.^12^ IMPT has been shown to have superior normal tissue sparing with less treatment-related toxic effects compared with IMRT in the treatment of several head and neck cancer subtypes, such as oropharyngeal cancer (OPC),^13,14,15^ salivary gland tumors,^16^ and sinonasal cancer.^17,18^

However, there is a paucity of data regarding the efficacy and toxicity profile of proton therapy for NPC, especially comparing IMPT with IMRT in the setting of newly diagnosed nonmetastatic NPC. This is likely because of the sporadic incidence of NPC in the Western world, where most proton centers are operating, and the lack of proton centers in the endemic regions.^19^ In the meantime, there is a compelling need for clinical evidence regarding the role of proton therapies, such as IMPT, in the management of NPC, as increasing numbers of new proton centers are opening or undergoing development worldwide. In this study, we examined a cohort of patients with nonmetastatic NPC who were treated with curative-intent RT to compare the toxicity profiles and oncologic outcomes of IMPT vs IMRT.

## Methods

### Patient Cohort

We retrospectively reviewed all consecutive adult patients (≥18 years) with newly diagnosed nonmetastatic NPC who were treated with chemoradiotherapy or RT alone at Memorial Sloan Kettering Cancer Center (MSKCC) between January 2016 and December 2019. This study was approved by the MSKCC institutional review board, and a waiver of informed consent was granted because of the retrospective nature of the study. This study follows the Strengthening the Reporting of Observational Studies in Epidemiology (STROBE) reporting guidelines for observational cohort studies. 

We excluded patients who received palliative RT or who had no follow-up after the completion of RT. IMPT was generally offered as an alternative to IMRT off trial or was necessitated for large tumors when IMRT could not be safely delivered. The reasons for patients not receiving IMPT included patient’s preference for IMRT as the standard of care; insurance denial of IMPT; and logistic issues for daily visit to the proton treatment center, which is located approximately 50 miles away from the MSKCC campus.

Details of pretreatment evaluation, radiation techniques, chemotherapy regimen, and follow-ups are described in the eMethods in the Supplement. Briefly, after appropriate pretreatment workup per the National Comprehensive Cancer Network (NCCN) guideline for NPC, patients with American Joint Committee on Cancer (AJCC) eighth edition stage I disease were treated with RT alone, while most patients with stage II to stage IVA disease were treated with concurrent chemoradiotherapy followed by adjuvant chemotherapy. Radiation dose to the gross tumor volume, high-risk anatomic sites, and low-risk anatomic sites were 69.96, 56 to 59.4, and 54.12 Gray equivalent (GyE), respectively, in 33 fractions or 70, 59 to 63, and 56 GyE, respectively, in 35 fractions. Concurrent chemotherapy was administered with cisplatin, either weekly (dose, 40mg/m^2^) for as many as 7 cycles or every 3 weeks (dose, 100mg/m^2^) for as many as 3 cycles. Induction chemotherapy regimen was administered at the discretion of treating medical oncologist. All patients were seen by the radiation oncologist weekly during treatment for toxic effects evaluation. Patients were subsequently followed up at 8 to 12 weeks after the completion of RT, then at 3-month intervals for the first 2 years, then every 6 to 12 months, with appropriate interval imaging studies and physical examinations.

### Data Collection

Clinicopathologic data were prospectively curated in a departmental registry. Treatment-related AEs were prospectively captured by standardized on-treatment and follow-up visit notes and verified by 2 of us (X.L. and S.K.). Treatment-related AEs were graded using Common Terminology Criteria for Adverse Events (CTCAE) version 4.0 (version 5.0 since April 2018). AEs occurring within 90 days after radiation treatment completion were considered acute, while those occurring after the 90-day period were considered late AEs. Radiation treatment records and oncologic outcomes were manually abstracted from electronic medical records (X.L. and S.K.) using a uniform data abstraction form. Local or regional failures were confirmed by both imaging studies and tissue biopsies. Locoregional failure-free survival (LRFS), progression-free survival (PFS), and overall survival (OS) were calculated from the start of RT until the occurrence of events or censoring. The end of the follow-up period was March 31, 2021.

### Statistical Analysis

The association of demographic characteristics, clinical characteristics, and treatment factors with treatment modality (IMPT vs IMRT) were assessed using χ^2^ test or Fisher exact test for categorial covariates and Wilcoxon rank sum test or Kruskal-Wallis test for continuous variable. The ordinal distribution of graded AEs was compared between IMPT vs IMRT groups using the Cochran-Armitage trend test. The association between binary toxic effect outcomes (development of any acute or chronic grade ≥2 or grade ≥3 AEs) and relevant clinical factors were evaluated by univariable logistic regression. Multivariable logistic regression models were then constructed with relevant covariates if the *P* value was less than .20. Multicollinearity was assessed by variance inflation factors testing.

Median follow-up time was determined by reverse Kaplan-Meier method. Cumulative incidence of LRF was calculated using death as a competing risk and compared between IMPT vs IMRT groups using the Fine-Gray method. The Kaplan-Meier method was used to generate and compare LRFS, PFS, and OS curves between IMPT vs IMRT groups with log-rank test. A multivariable Cox proportional hazard model was used to calculate hazard ratios (HRs) based on a panel of covariates determined a priori, including RT modality, sex, age, smoking history, Karnofsky Performance Score (KPS), Epstein-Barr virus (EBV) status, and disease stage.

A 1:1 propensity score matching (PSM) was performed to address potential biases due to the retrospective nature of the study when analyzing the oncologic outcomes. The bootstrapping method was used with 1:1 nearest-neighbor matching using caliper of 10%. Matching was performed with variables found to be significantly associated with receipt of IMPT on univariable regression analysis, including T4 disease, nonsmoking status, and receipt of high-dose cisplatin. Survival analyses were repeated in the matched cohort, adjusting for propensity score quintile. All tests were 2-sided, and *P* < .05 was considered statistically significant. All analyses were performed using R software version 4.0 (R Project for Statistical Computing)

## Results

### Patient Characteristics

We identified 77 eligible patients (25 [32.5%] women; 52 [67.5%] men; median [interquartile range {IQR}] age, 48.7 [42.2-60.3] years) with newly diagnosed nonmetastatic NPC who received curative intent treatment with RT alone (6 [7.8%]) or chemoradiotherapy (71 [92.2%]). Twenty-eight patients (36.4%) were treated with IMPT, and 49 patients (63.6%) were treated with IMRT. Table 1 summarizes the clinical characteristics by RT modality (ie, IMPT vs IMRT). Overall, the 2 groups appeared to be balanced in age, sex, smoking history, performance status, AJCC staging, EBV status, World Health Organization (WHO) subtype, and use of chemotherapy. Most patients (69 [89.6%]) had excellent performance status at the beginning of RT (KPS 90-100). Most patients had EBV-positive disease (69 [89.6%]) and WHO type 2b (nonkeratinizing undifferentiated carcinoma [NKUC], endemic subtype) NPC (69 [89.6%]). Significantly more patients in the IMPT group received high-dose cisplatin (16 [57.1%] vs 12 [24.5%]; *P* = .004) for concurrent chemotherapy. Although not statistically significant, numerically more patients in the IMPT group had T4 disease (8 [28.6%] vs 6 [12.2%]; *P* = .14), had human papillomavirus (HPV)–positive NPC (4 [14.3%] vs 1 [2.0%]; *P* = .06), and received induction chemotherapy (6 [21.4%] vs 3 [6.1%]; *P* = .06) compared with the IMRT group.

**Table 1.** Baseline Clinical Characteristics of Patients by Radiation Modality

Characteristic	Patients, No. (%)		*P* value^a^
	IMPT (n = 28)	IMRT (n = 49)	
Age at RT, median (IQR), y	45.9 (42.0-59.6)	49.5 (42.6-60.9)	.68
Sex			
Male	19 (67.9)	33 (67.3)	.96
Female	9 (32.1)	16 (32.7)	
Smoking history			
No	21 (75.0)	28 (57.1)	.12
Yes	7 (25.0)	21 (42.9)	
KPS			
100	2 (7.1)	6 (12.2)	.77
90	22 (78.6)	38 (77.6)	
80	4 (14.3)	5 (10.2)	
T stage			
Tx	1 (3.6)	0	.09
T1	7 (25.0)	21 (42.9)	
T2	3 (10.7)	8 (16.3)	
T3	9 (32.1)	14 (28.6)	
T4	8 (28.6)	6 (12.2)	
N stage			
N0	7 (25.0)	5 (10.2)	.33
N1	10 (35.7)	23 (46.9)	
N2	10 (35.7)	18 (36.7)	
N3	1 (3.6)	3 (6.1)	
AJCC eighth edition stage			
I	4 (14.3)	3 (6.1)	.39
II	4 (14.3)	13 (26.5)	
III	11 (39.3)	25 (51.0)	
IVA	9 (32.1)	8 (16.3)	
EBV status			
Positive	24 (85.7)	45 (91.8)	.28
Negative or unknown	4 (14.3)	4 (8.2)	
HPV status			
Positive	4 (14.3)	1 (2.0)	.06
Negative or unknown	24 (85.7)	48 (98.0)	
WHO Classification			
Type 1, KSCC	0	1 (2.0)	.45
Type 2a, NKDC	3 (10.7)	3 (6.1)	
Type 2b, NKUC	24 (85.7)	45 (91.8)	
Basaloid	1 (3.6)	0	
Treatment regimen			
RT alone	3 (10.7)	3 (6.1)	.66
Chemoradiotherapy	25 (89.3)	46 (93.9)	
Induction chemotherapy	6 (21.4)	3 (6.1)	.06
Concurrent chemotherapy	25 (89.3)	46 (93.9)	.66
Type of concurrent chemotherapy			
High-dose cisplatin	16 (57.1)	12 (24.5)	.004
Weekly cisplatin	12 (42.9)	37 (75.5)	
Adjuvant chemotherapy	11 (39.3)	26(53.1)	.34
RT dose, median (IQR), GyE	70.0 69.96-70.0)	69.96 (69.96-69.96)	>.99

Abbreviations: AJCC, American Joint Committee on Cancer; EBV, Epstein-Barr virus; GyE, Gray equivalent; HPV, human papillomavirus; IMPT, intensity-modulated proton therapy; IMRT, intensity-modulated radiation therapy; IQR, interquartile range; KPS, Karnofsky Performance Score; KSCC, keratinizing squamous cell carcinoma; NKDC, nonkeratinizing differentiated carcinoma; NKUC, nonkeratinizing undifferentiated carcinoma; RT, radiotherapy; WHO, World Health Organization.

^a^ Categorical variables were assessed by χ^2^ test or Fisher exact test. Continuous variables were compared by Wilcoxon rank sum test.

### Treatment Characteristics and Adverse Events

All patients received biologically equivalent doses of radiation (70 GyE) to the primary tumor and grossly involved lymph nodes, with comparable subclinical dose (50-60 GyE) to regions at risk of microscopic involvement. Radiation doses to the major organs at risks are summarized by RT modality (IMPT vs IMRT) in eTable 1 in the Supplement. IMPT treatment was associated with significantly lower mean oral cavity dose (median [IQR], 15.4 [12.3-21.4] GyE vs 32.8 [30.2-37.1] GyE; *P* < .001), lower mean larynx dose (median [IQR], 16.0 [12.9-20.2] GyE vs 29.6 [22.8-33.1] GyE; *P* < .001), and lower mean parotid gland dose (median [IQR], 22.5 [19.8-25.6] GyE vs 25.2 [23.0-26.5] GyE; *P* = .01) compared with patients treated with IMRT.

Complete records of acute AEs were available for all 77 patients. IMPT treatment appeared to be associated with a significant trend of lower grades of specific acute AEs, including dysphagia, fatigue, xerostomia, dysgeusia, oral mucositis, weight loss, and hoarseness (eTable 2 in the Supplement). Overall, 19 of 28 patients (67.9%) receiving IMPT and 46 of 49 patients (93.9%) receiving IMRT developed any grade 2 or higher acute AE. Univariable analysis was then carried out to investigate potential associations between clinically relevant factors (ie, RT modality, sex, age, smoking history, KPS, tumor staging, use of and type of concurrent chemotherapy) and the development of any grade 2 or higher acute AEs (Table 2). On univariable analysis, IMPT treatment was associated with significantly lower likelihood of developing grade 2 or higher acute AEs compared with IMRT treatment (odds ratio [OR], 0.14; 95% CI, 0.03-0.52; *P* = .006). On multivariable logistic regression analysis, IMPT treatment remained the only factor that was associated with significantly lower likelihood of developing grade 2 or higher acute AEs (OR, 0.15; 95% CI 0.03-0.60; *P* = .01). Grade 3 or higher acute AEs were only observed in 3 of 28 patients (10.7%) receiving IMPT, while 11 of 49 patients (22.4%) receiving IMRT developed grade 3 or higher acute AEs (most common were dysphagia, oral mucositis, weight loss, nausea). On multivariable analysis, IMPT was not associated with significantly lower likelihood of developing any grade 3 or higher acute AE compared with IMRT (OR, 0.21; 95% CI, 0.01-1.21; *P* = .15). No other clinicopathological factors were found to be associated with significantly lower likelihood of grade 3 or higher acute AEs. No grade 4 or 5 acute AEs were observed in this study.

**Table 2.** Univariable and Multivariable Analyses for Factors Associated With the Development of Grade 2 or Higher Acute Adverse Events

Variable	Univariable		Multivariable^a^	
	OR (95% CI)	*P* value	OR (95% CI)	*P* value
RT modality				
IMRT	1 [Reference]	NA	1 [Reference]	NA
IMPT	0.14 (0.03-0.52)	.006	0.15 (0.03-0.60)	.01
Sex				
Female	1 [Reference]	NA	NA	NA
Male	0.65 (0.13-2.44)	.55	NA	NA
Age	0.96 (0.91-1.02)	.18	0.97 (0.91-1.02)	.24
Smoking history				
No	1 [Reference]	NA	NA	NA
Yes	1.87 (0.50-9.05)	.38	NA	NA
KPS	1.04 (0.91-1.19)	.57	NA	NA
T stage				
T3-T4	1 [Reference]	NA	1 [Reference]	NA
T1-T2	3.96 (1.07-19.12)	.05	2.90 (0.70-14.92)	.16
N stage				
N2-N3	1 [Reference]	NA	NA	NA
N0-N1	1.01 (0.27-3.49)	>.99	NA	NA
AJCC eighth edition stage				
III-IVA	1 [Reference]	NA	NA	NA
I-II	2.56 (0.61-17.60)	.25	NA	NA
Concurrent chemotherapy				
No	1 [Reference]	NA	NA	NA
Yes	3.05 (0.39-17.95)	.23	NA	NA
Type of concurrent chemotherapy				
Weekly cisplatin	1 [Reference]	NA	NA	NA
High dose cisplatin	0.51 (0.14-1.81)	.29	NA	NA

Abbreviations: AJCC, American Joint Committee on Cancer; IMPT, intensity-modulated proton therapy; IMRT, intensity-modulated radiation therapy; KPS, Karnofsky Performance Score; NA, not applicable; OR, odds ratio; RT, radiotherapy.

^a^ Variables missing *P* values on the multivariable analysis did not meet the inclusion criterion (*P* < .20 on univariable analysis); therefore, they were not included in the multivariable logistic regression model.

Records of chronic AEs for 2 patients (7.1%) in the IMPT group were unavailable because of loss to follow-up after the first post-RT visit. Five of 26 patients (19.2%) receiving IMPT and 14 of 49 patients (28.6%) receiving IMRT developed at least 1 grade 2 or higher chronic AE (most common were hearing impairment, xerostomia, fatigue, dysphagia, dysgeusia). Only age was significantly associated with lower likelihood of developing any grade 2 or higher chronic AE on univariable analyses (OR, 1.07; 95% CI, 1.02-1.13; *P* = .01). Only 1 patient (3.8%) receiving IMPT developed grade 3 or higher chronic AEs (percutaneous endoscopic gastrostomy [PEG] tube placed for severe dysphagia) compared with 8 patients (16.3%) in the IMRT group (2 PEG tube dependence, 2 severe hearing impairment, 2 severe weight loss, 2 severe oral pain) (OR, 0.21; 95% CI, 0.01-1.21; *P* = .15). After adjusting for covariates on multivariable logistic regression, IMPT was not associated with significantly lower likelihood of developing any grade 3 or higher chronic AE compared with IMRT (OR, 0.16; 95% CI, 0.01-1.03; *P* = .11). Specifically, IMPT treatment was not associated with lower likelihood of PEG tube dependency in the chronic setting compared with IMRT treatment (2 of 26 [7.7%] vs 3 of 49 [6.1%]; OR, 1.18; 95% CI, 0.15-7.56; *P* = .86). No grade 4 or 5 chronic AEs were observed.

### Survival Outcomes

The median (IQR) follow-up time for the entire cohort, IMPT group, and IMRT group were 30.3 (17.9-41.5) months, 18.7 (13.5-30.0) months, and 37.0 (26.0-44.0) months, respectively. After excluding the 2 patients in the IMPT group with early loss to follow-up (not included in the analysis for chronic AEs), the median (IQR) follow-up time for the IMPT group increased to 23.0 (14.6-30.2) months.

**Figure 1.**
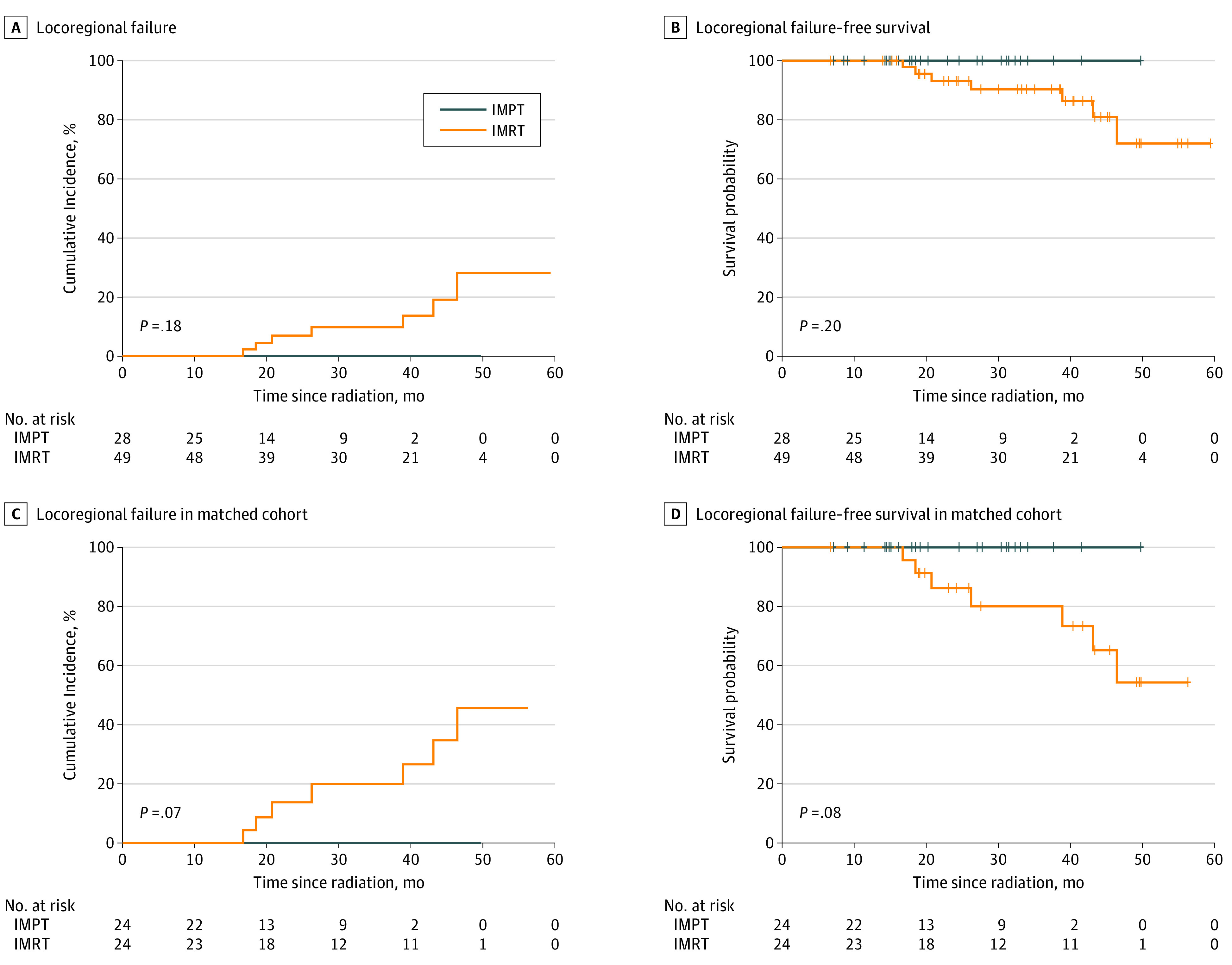
Cumulative Incidence of Locoregional Failure and Locoregional Failure–Free Survival by Radiotherapy Modality IMPT indicates intensity-modulated proton therapy; IMRT, intensity-modulated radiation therapy.

No LRF was observed in the IMPT group, while 5 local failures and 4 regional failures occurred in 7 patients (14.3%) from the IMRT group. When death was included as a competing risk, the cumulative incidences of LRF in IMPT vs IMRT group was estimated to be 0.0% vs 4.4% (95% CI, 3.5%-5.3%) at 20 months and 0.0% vs 9.6% (95% CI, 8.3%-10.9%) at 30 months, which was not statistically different (*P* = .18) (Figure 1A). On multivariable Fine-Gray regression analysis, IMPT was found to be associated with decreased likelihood of LRF (IMPT vs IMRT; HR, 0.00; 95% CI, 0.00-0.00; *P* < .001), while EBV-positive disease was associated with increased likelihood of LRF compared with EBV-negative disease (HR, 10 855.41; 95% CI, 777.48-151 559.66; *P* < .001). There was no significant difference in LRFS between the IMPT and IMRT groups (Figure 1B). On multivariable Cox proportional hazard analyses, no individual clinical factor was found to be significantly associated with LRFS (eTable 3 in the Supplement).

**Figure 2.**
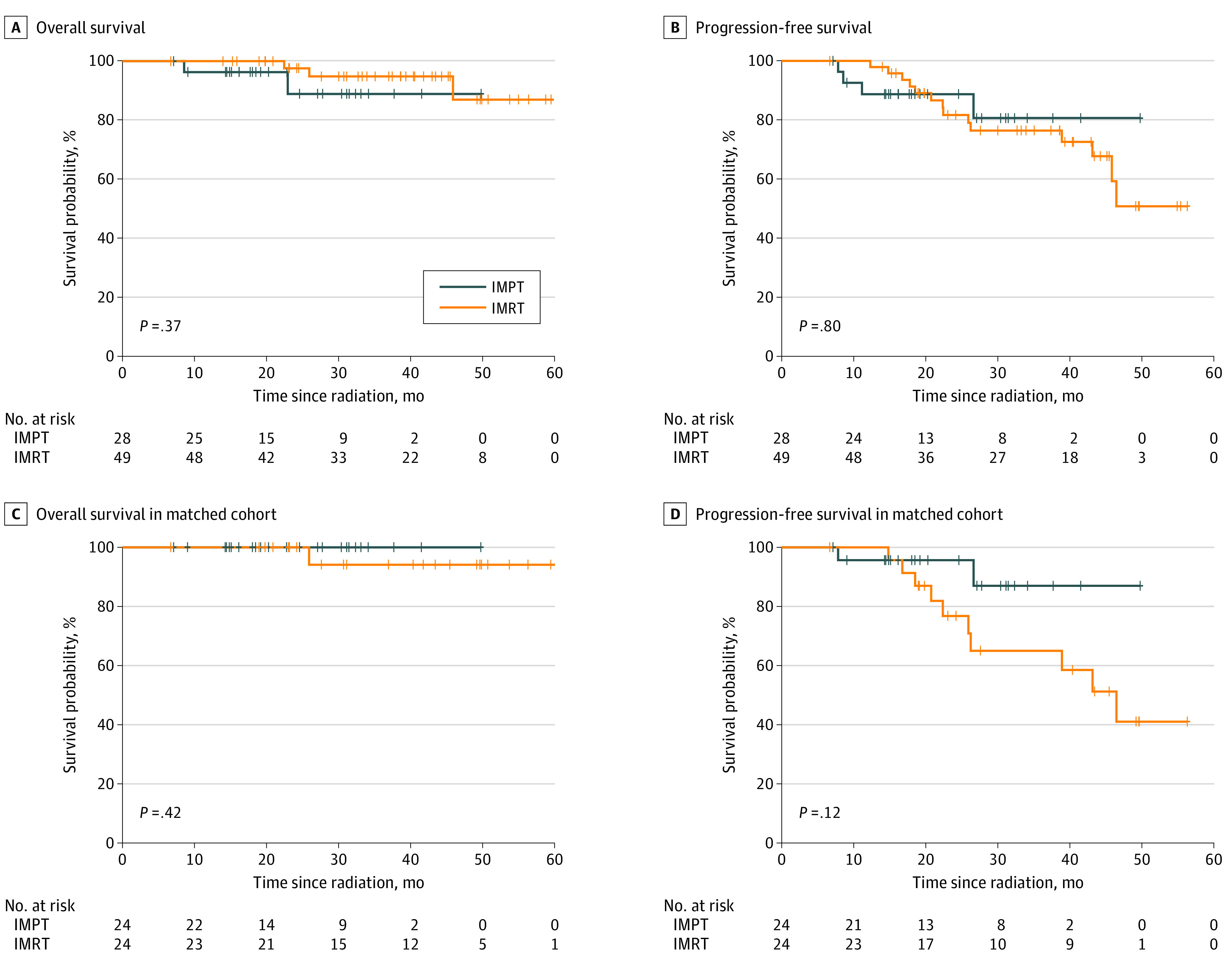
Overall Survival and Progression-Free Survival by Radiotherapy Modality IMPT indicates intensity-modulated proton therapy; IMRT, intensity modulated radiation therapy.

There was no significant difference in OS between IMPT and IMRT groups (HR, 2.29; 95% CI, 0.36-14.67; *P* = .37) (Figure 2A). Smoking history was not associated with worse OS (smoking vs nonsmoking: HR, 8.73; 95% CI, 0.97-78.24; *P* = .05), while EBV-positive status was associated with better OS vs EBV-negative disease or unknown status (HR, 0.05; 95% CI, 0.01-0.39; *P* = .004) on univariable analyses (Table 3). No clinical factor was significantly associated with OS on multivariable analyses. PFS was not significantly different between IMPT vs IMRT groups (HR, 0.86; 95% CI, 0.28-2.68; *P* = .80) (Figure 2B). On multivariable analyses, male sex appeared to be associated with worse PFS (male vs female: HR, 5.84; 95% CI, 1.19-28.72; *P* = .03), while early T stage and EBV-positive status were associated with better OS (T1-2 vs T3-4: HR, 0.20; 95% CI, 0.06-0.66; *P* = .008; EBV-positive disease vs negative or unknown: HR, 0.03; 95% CI, 0.00-0.27; *P* = .002) (Table 3).

**Table 3.** Univariable and Multivariable Analyses for Overall Survival and Progression-Free Survival

Variable	Overall survival				Progression-free survival			
	Univariable		Multivariable^a^		Univariable		Multivariable^a^	
	HR (95% CI)	*P* value	HR (95% CI)	*P* value	HR (95% CI)	*P* value	HR (95% CI)	*P* value
RT modality								
IMRT	1 [Reference]	NA	1 [Reference]	NA	1 [Reference]	NA	1 [Reference]	NA
IMPT	2.29 (0.36-14.67)	.38	9.21 (0.26-328.37)	.22	0.86 (0.28-2.68)	.80	0.71 (0.19-2.68)	.62
Sex								
Female	1 [Reference]	NA	1 [Reference]	NA	1 [Reference]	NA	1 [Reference]	NA
Male	1.76 (0.20-15.79)	.61	5.93 (0.12-287.48)	.37	1.93 (0.63-5.89)	.25	5.84 (1.19-28.72)	.03
Age	1.08 (0.99-1.19)	.10	1.05 (0.92-1.20)	.48	1.03 (0.98-1.07)	.23	1.01 (0.96-1.06)	.72
Smoking history								
No	1 [Reference]	NA	1 [Reference]	NA	1 [Reference]	NA	1 [Reference]	NA
Yes	8.73 (0.97-78.24)	.05	24.43 (0.86-698.07)	.06	2.29 (0.91-5.79)	.08	2.21 (0.73-6.71)	.16
KPS	0.92 (0.76-1.10)	.34	NA	NA	0.95 (0.86-1.04)	.28	0.97 (0.89-1.07)	.60
T stage								
T3-T4	1 [Reference]	NA	1 [Reference]	NA	1 [Reference]	NA	1 [Reference]	NA
T1-T2	1.09 (0.18-6.62)	.92	0.33 (0.02-5.32)	.43	0.40 (0.15-1.04)	.06	0.20 (0.06-0.66)	.008
N stage								
N2-N3	1 [Reference]	NA	NA	NA	[Reference]	NA	NA	NA
N0-N1	0.98 (0.16-5.92)	.99	NA	NA	1.08 (0.42-2.80)	.87	NA	NA
AJCC eighth edition stage								
III-IVA	1 [Reference]	NA	NA	NA	1 [Reference]	NA	NA	NA
I-II	1.35 (0.22-8.07)	.74	NA	NA	0.80 (0.28-2.25)	.67	NA	NA
EBV status								
Negative or unknown	1 [Reference]	NA	1 [Reference]	NA	1 [Reference]	NA	1 [Reference]	NA
Positive	0.05 (0.01-0.39)	.004	0.04 (0.00-2.25)	.12	0.14 (0.04-0.51)	.003	0.03 (0.00-0.27)	.002
HPV status								
Negative or unknown	1 [Reference]	NA	NA	NA	1 [Reference]	NA	NA	NA
Positive	42.95 (3.81-483.75)	.002	NA	NA	22.67 (4.35-118.22)	<.001	NA	NA
Concurrent chemotherapy								
No	1 [Reference]	NA	NA	NA	1 [Reference]	NA	NA	NA
Yes	0.24 (0.02-2.27)	.21	NA	NA	0.41 (0.09-1.83)	.24	NA	NA
Type of concurrent chemo								
Weekly cisplatin	1 [Reference]	NA	NA	NA	1 [Reference]	NA	NA	NA
High-dose cisplatin	0.76 (0.08-7.17)	.81	NA	NA	1.79 (0.68-4.73)	.24	NA	NA

Abbreviations: AJCC, American Joint Committee on Cancer; EBV, Epstein-Barr virus; HR, hazard ratio; HPV, human papillomavirus; IMPT, intensity-modulated proton therapy; IMRT, intensity-modulated radiation therapy; KPS, Karnofsky Performance Score; NA, not applicable; RT, radiotherapy.

^a^ Multivariable Cox proportional hazard model included a panel of covariates determined a priori, as follows: RT modality, sex, age, smoking history, KPS, EBV status, and disease stage.

Considering that HPV-related NPC is associated with worse prognosis than EBV-related NPC^20^ as well as other potential selection biases introduced by the retrospective nature of the study, we performed propensity score matching in patients with HPV-negative and EBV-positive disease using factors associated with higher likelihood of receiving IMPT, including T4 disease, nonsmoking status, and receipt of high-dose cisplatin. A well-matched cohort of 48 patients (24 IMPT vs 24 IMRT) was generated with balanced clinical characteristics (eTable 4 in the Supplement).

The median (IQR) follow-up times for the IMPT group and IMRT group in the matched cohort were 23.0 (13.5-30.2) months and 38.5 (22.7-48.0) months, respectively. In the matched cohort, the cumulative incidences of LRF in IMPT vs IMRT group with death as competing risk were estimated to be 0.0% vs 8.7% (95% CI, 7.0%-10.4%) at 20 months and 0.0% vs 19.5% (95% CI, 17.0%-22.1%) at 30 months (*P* = .07) (Figure 1C). The 2-year LRFS was 100% (95% CI, 100%-100%) in the IMPT group and 86.2% (95% CI, 72.8%-100%) in the IMRT group (*P* = .08) (Figure 1D). Smoking history was found to be associated with poor LRFS (smoking vs nonsmoking: HR, 63.37; 95% CI, 3.25-1236.13; *P* = .006) on multivariable analyses (eTable 3 in the Supplement). No patient died within 2 years of RT in the matched cohort. Three-year OS was 100% (95% CI, 100%-100%) in the IMPT group and 94.1% (95% CI, 83.6%-100%) in the IMRT group (*P* = .42) (Figure 2C). The 2-year PFS in the matched cohort was 95.7% (95% CI, 87.7%-100%) in the IMPT group vs 76.7% (95% CI, 60.7%-97.0%) in the IMRT group (HR, 0.31; 95% CI, 0.07-1.47; *P* = .14) (Figure 2D). Smoking history was the only significant factor associated with PFS on multivariable analysis (smoking vs nonsmoking: HR, 6.33; 95% CI, 1.16-34.57; *P* = .03). Because no LRF or death occurred in the IMPT group from the matched cohort, the 95% CI for the hazard ratio of IMPT vs IMRT for LRFS and OS could not be calculated.

## Discussion

To our knowledge, this study represented the largest comparative analysis of curative intent IMPT vs IMRT as the primary RT modality for nonmetastatic NPC, while the previously largest published series included only 10 patients receiving IMPT.^21^ Other published series regarding proton therapy in NPC either used an outdated proton RT technique or mainly focused on re-irradiation for recurrent NPC, where the treatment toxic effects and oncologic outcomes are expected to be significantly different than in untreated nonmetastatic NPC.^22,23^ In the meantime, more than 30 proton treatment centers are being constructed or are in planning phase in Asia, where 81% of global NPC cases are diagnosed.^24,25^ Therefore, our study is providing timely and valuable evidence regarding IMPT as the primary RT modality for curative treatment of NPC.

The key findings of this study showed that IMPT treatment delivered a significantly reduced radiation dose to normal tissues, including the oral cavity, larynx, and parotid glands, which could explain the association between IMPT treatment and lower risk of specific acute AEs, such as oral mucositis, dysphagia, dysgeusia, dry mouth, hoarseness, and weight loss. The dosimetry advantage of IMPT vs IMRT in NPC treatment has been reproducibly demonstrated over the years; therefore, it was not the focus of this study.^26,27^ Probably more clinically meaningful, IMPT treatment was associated with significantly lower risk of developing any grade 2 or higher acute AEs. Because grade 2 or higher AEs usually mean symptomatic AEs requiring medical intervention or affecting patient’s ability to perform instrumental activities of daily living,^21^ the potential savings on health care resources for managing AEs and the improvement on patient’s quality of life associated with IMPT could be immense, albeit not directly addressed by this study.

In a similar study by Holliday et al^21^ comparing 10 patients with NPC treated by IMPT to 20 matched patients with NPC treated by IMRT, the authors found lower mean doses to the oral cavity, brainstem, whole brain, and mandible by IMPT. They also found that increased mean dose to the oral cavity was associated with a higher rate of PEG tube placement. Of note, the rate of PEG tube placement in their study was as high as 65% in the IMRT group. In contrast, PEG tube placement was equally rare (<10%) in both the IMPT and IMRT groups in our cohort, likely reflecting the different practice preferences in the management or failure to thrive due to treatment related toxic effects, such as pain, mucositis, and dysphagia. At our institution, we engage a supportive care team early in the treatment course for optimal pain management and psychosocial support to avoid PEG tube placement unless absolutely necessary.

The cumulative incidences of acute and chronic grade 3 AEs were both numerically lower in the IMPT group than the IMRT group, although the differences did not reach statistical significance. Nevertheless, with a median follow-up close to 2 years in the IMPT group for chronic AEs, the absence of any severe (ie, grade ≥3) toxic effects, such as temporal lobe necrosis, osteoradionecrosis, or radiation-induced optic neuropathy, is encouraging. Longer follow-up is necessary and has been planned considering that late toxic effects may occur more than 3 to 5 years after proton RT.^10^

There was no LRF in the IMPT group compared with 7 LRFs in the IMRT group, although the difference was not significant. Improving the current cohort to larger size with longer follow-up time for data to mature with more events may potentially demonstrate a significant association between IMPT and better locoregional control. PFS and OS were similar between the IMPT and IMRT groups. Adjusted for potential biases to our best effort, these findings persisted in the propensity score–matched subanalysis. Both groups showed quite favorable OS outcomes in comparison with historical series,^2,4^ highlighting the advancement in the management of NPC during the past few decades. In the absence of randomized data in the foreseeable future, we believe our propensity score–matched analysis in a contemporary cohort of patients with nonmetastatic NPC provides valuable evidence for the global oncology community regarding the toxicity profile and clinical efficacy of IMPT for nonmetastatic NPC.

### Limitations

This study has limitations. The major limitation is the retrospective nature, which limits the strength of conclusions on potential associations, despite our best effort to address potential selection biases with multivariable analyses and propensity score matching. Another limitation is the small number of patients in the IMPT group, limiting meaningful subset analyses to guide patient selection. Further limitations include imbalanced median follow-up time in the IMPT group (23.0 months) vs the IMRT group (37.0 months), which was likely a result of increasingly more patients being treated by IMPT in recent years. This could be problematic given that LRF and late complications may occur after 2 years. Meanwhile, an important cofounder, which is socioeconomic status, including insurance status, was not captured in this study, which could be associated with both the affordability of IMPT and survival outcomes of the same patient. Finally, patient-reported outcomes were only available for a fraction of patients, and incomplete data precluded conclusive analysis of these meaningful outcome measures.

## Conclusions

In this study, curative-intent RT with IMPT for nonmetastatic NPC was associated with significantly reduced acute toxicity burden in comparison with IMRT, with rare late complications and excellent oncologic outcomes, including 100% locoregional control at 2 years. Prospective studies are warranted to direct optimal patient selection for IMPT in nonmetastatic NPC, especially in endemic regions where IMPT may become readily available in the coming years.

## Notes

Supplement. **eTable 1. **Dose to Organs at Risk for Patients Treated With IMPT vs IMRT **eTable 2. **Comparison of Specific Acute Adverse Events for Patients Treated With IMPT vs IMRT **eTable 3. **Univariable and Multivariable Analyses for Locoregional Failure-Free Survival Before and After Matching **eTable 4. **Baseline Clinical Characteristics of Patients by Radiation Modality in Propensity Score–Matched Cohort **eTable 5. **Univariable and Multivariable Analyses for Progression-Free Survival in Propensity Score–Matched Cohort **eMethods. **Details of Pretreatment Evaluation, Radiation Technique, Chemotherapy Regimen, and Follow-up Strategy
