# Error in Article Information

**DOI:** 10.1001/jamanetworkopen.2021.35629

**Published:** 2021-10-20

In the Original Investigation titled “Toxicity Profiles and Survival Outcomes Among Patients With Nonmetastatic Nasopharyngeal Carcinoma Treated With Intensity-Modulated Proton Therapy vs Intensity-Modulated Radiation Therapy,”^1^ published June 18, 2021, there was an error in the Article Information. The Funding/Support and Role of the Funder/Sponsor sections were missing. This article has been corrected.^1^
