# Supplementary material for: Toxicity Profiles and Survival Outcomes Among Patients With Nonmetastatic Nasopharyngeal Carcinoma Treated With Intensity-Modulated Proton Therapy vs Intensity-Modulated Radiation Therapy
Source: JAMA Netw Open. 2021 Jun 18;4(6):e2113205. doi: 10.1001/jamanetworkopen.2021.13205 (PMC8214161; doi:10.1001/jamanetworkopen.2021.13205)
Supplement: Supplement. — eTable 1. Dose to Organs at Risk for Patients Treated With IMPT vs IMRT eTable 2. Comparison of Specific Acute Adverse Events for Patients Treated With IMPT vs IMRT eTable 3. Univariable and Multivariable Analyses for Locoregional Failure-Free Survival Before and After Matching eTable 4. Baseline Clinical Characteristics of Patients by Radiation Modality in Propensity Score–Matched Cohort eTable 5. Univariable and Multivariable Analyses for Progression-Free Survival in Propensity Score–Matched Cohort eMethods. Details of Pretreatment Evaluation, Radiation Technique, Chemotherapy Regimen, and Follow-up Strategy [file jamanetwopen-e2113205-s001.pdf]

Supplemental Online Content

Li X, Kitpanit S, Lee A, et al. Toxicity profiles and survival outcomes among patients with nonmetastatic nasopharyngeal carcinoma treated with intensity-modulated proton therapy vs intensity-modulated radiation therapy. *JAMA Netw Open*. 2021;4(6):e2113205. doi:10.1001/jamanetworkopen.2021.13205

eTable 1. Dose to Organs at Risk for Patients Treated with IMPT vs IMRT
eTable 2. Comparison of Specific Acute Adverse Events for Patients Treated with IMPT vs IMRT
eTable 3. Univariable and Multivariable Analyses for Locoregional Failure-Free Survival Before and After Matching
eTable 4. Baseline Clinical Characteristics of Patients by Radiation Modality in Propensity Score–Matched Cohort
eTable 5. Univariable and Multivariable Analyses for Progression-Free Survival in Propensity Score–Matched Cohort
eMethods. Details of Pretreatment Evaluation, Radiation Technique, Chemotherapy Regimen, and Follow-up Strategy

This supplemental material has been provided by the authors to give readers additional information about their work.

© 2021 Li X et al. *JAMA Network Open.*

eTable 1. Dose to Organs at Risk for Patients Treated with IMPT vs IMRT

Dosimetry Parameter[a]	IMPT (n=28), GyE	IMRT (n=49), GyE	P value[b]
Oral Cavity (Mean)	15.4 (12.3, 21.4)	32.8 (30.2, 37.1)	**<.001**
Larynx (Mean)	16.0 (12.9, 20.2)	29.6 (22.8, 33.1)	**<.001**
Larynx (Max)	53.8 (48.2, 58.4)	57.6 (52.2, 62.7)	.06
Parotid Gland (Mean)	22.5 (19.8, 25.6)	25.2 (23.0, 26.5)	**.01**
Optic Chiasm (D0.05cc[c])	37.2 (27.1, 48.3)	43.1 (20.8, 52.2)	.64
Optic Nerve (D0.05cc)	48.6 (34.1, 55.2)	40.5 (24.9, 53.5)	.14
Brainstem (D0.05cc)	58.5 (55.6, 59.3)	59.0 (55.3, 59.8)	.27
Temporal Lobe (D2cc)	64.5 (58.5, 68.6)	59.4 (50.0, 61.6)	**.02**
Cochlea (Mean)	43.0 (41.1, 44.9)	43.8 (42.0, 46.2)	.47
Spinal Cord (D0.1cc)	44.3 (41.7, 48.4)	44.3 (42.9, 46.0)	.61

Abbreviations: IMPT, intensity-modulated proton therapy; IMRT, intensity modulated radiation therapy; GyE, Gray equivalent.

a. Statistics presented: median and interquartile range (IQR)
b. P value was obtained from Wilcoxon rank sum test
c. D*x*cc, the minimal dose to the highest irradiated *x* cm^3 of corresponding OARs.

© 2021 Li X et al. *JAMA Network Open.*

eTable 2. Comparison of Specific Acute Adverse Events for Patients Treated with IMPT vs IMRT

Toxicities		Acute Adverse Events[a] (%)				P value[b]
		None	Grade 1	Grade 2	Grade 3	
**Oral Pain**	IMPT	14.3	75.0	10.7	0	.66
	IMRT	12.2	69.4	18.4	0	
**Dysphagia**	IMPT	21.4	60.7	14.3	3.6	**.05**
	IMRT	10.2	42.9	42.9	4.1	
**Fatigue**	IMPT	25.0	64.3	10.7	0	**.02**
	IMRT	4.1	73.5	22.4	0	
**Xerostomia**	IMPT	50.0	42.9	7.1	0	**.002**
	IMRT	14.3	63.3	22.4	0	
**Dysgeusia**	IMPT	39.3	53.6	7.1	0	**.004**
	IMRT	12.2	57.1	30.6	0	
**Dermatitis**	IMPT	7.1	64.3	25.0	3.6	.45
	IMRT	10.2	46.9	40.8	2.0	
**Mucositis**	IMPT	10.7	64.3	21.4	3.6	**.03**
	IMRT	8.2	32.7	49.0	10.2	
**Weight Loss**	IMPT	39.3	25.0	35.7	0	**<.001**
	IMRT	4.1	36.7	49.0	10.2	
**Hoarseness**	IMPT	92.9	7.1	0	0	**.007**
	IMRT	63.3	32.7	4.1	0	
**Nausea**	IMPT	50.0	28.6	7.1	14.3	**.03**
	IMRT	30.6	55.1	12.2	2.0	

Abbreviations: IMPT, intensity-modulated proton therapy; IMRT, intensity modulated radiation therapy.

a. No grade 4 or 5 acute AEs occurred in the entire cohort.

b. P value was obtained from Chi-squre test for trend (Cochran-Armitage Test).

© 2021 Li X et al. *JAMA Network Open.*

eTable 3. Univariable and Multivariable Analyses for Locoregional Failure-Free Survival Before and After Matching

	**Before Matching (N=77)**				**After Matching (N=48)**			
	**Univariable**		**Multivariable[a]**		**Univariable**		**Multivariable[b]**	
**Variable**	**HR (95% CI)**	**P**	**HR (95% CI)**	**P**	**HR (95% CI)**	**P**	**HR (95% CI)**	**P**
RT Modality								
IMRT	Reference							
IMPT	0.00 (0.00-inf)	>.99			0.00 (0.00-inf)	>.99		
Sex								
Female	Reference							
Male	1.28 (0.25-6.61)	.77	1.17(0.20-6.82)	.86	1.74 (0.33-9.22)	.51	2.29(0.24-21.4)	.47
Age	1.00 (0.94-1.08)	.18	1.00 (0.92-1.08)	.98	1.00 (0.94-1.06)	.99	1.01 (0.93-1.10)	.84
Smoking History								
No	Reference							
Yes	1.77 (0.39-7.93)	.46	2.83 (0.55-14.7)	0.22	24.9 (2.27-273)	**.009**	63.4 (3.25-1236)	**.006**
KPS	1.06 (0.91-1.25)	.45	1.09 (0.93-1.28)	0.28	1.10 (0.92-1.31)	.31	1.29 (0.95-1.76)	.10
T-stage								
T3-T4	Reference							
T1-T2	0.44 (0.10-2.01)	.29	0.28 (0.05-1.54)	0.14	1.05 (0.23-4.81)	.95	0.28 (0.03-2.78)	.27
N-stage								
N2-N3	Reference							
N0-N1	1.73 (0.33-9.00)	.51			1.32 (0.25-6.88)	.74		
Stage (AJCC 8th)								
III-IVA	Reference							
I-II	0.76 (0.15-3.93)	.74			1.18 (0.23-6.12)	.84		
EBV Status								
Positive	Reference							
Negative/Unknown	0.00 (0.00-inf)	>.99						
Concurrent Chemo								

© 2021 Li X et al. *JAMA Network Open.*

No	Reference							
Yes	0.23 (0.02-2.23)	.20			0.17 (0.02-1.63)	.12		
Type of Concurrent Chemo								
Weekly cisplatin	Reference							
High dose cisplatin	1.38 (0.26-7.37)	.71			0.91 (0.17-4.92)	.91		

a. Multivariable Cox proportional hazard model included a panel of covariates determined *a priori*: radiotherapy modality, sex, age, smoking history, KPS, EBV status and disease stage. Radiotherapy modality and EBV status were removed from the model due to event=0 in one group.

b. Multivariable Cox proportional hazard model included a panel of covariates determined *a priori*: radiotherapy modality, sex, age, smoking history, KPS and disease stage. Radiotherapy modality was removed from the model due to event=0 in one group.

© 2021 Li X et al. *JAMA Network Open.*

eTable 4. Baseline Clinical Characteristics of Patients by Radiation Modality in Propensity Score–Matched Cohort

	No. (%)		
Characteristics	**IMPT (n=24)**	**IMRT (n=24)**	**P value[a]**
Age at RT, median (IQR), yr	44.6 (41.1,58.7)	53.9 (35.9,61.5)	.42
Sex			>.99
Male	17 (70.8)	17 (70.8)	
Female	7 (29.2)	7 (29.2)	
Smoking History			>.99
No	18 (75.0)	18 (75.0)	
Yes	6 (25.0)	6 (25.0)	
KPS			>.99
100	2 (8.3)	2 (8.3)	
90	19 (79.2)	19 (79.2)	
80	3 (12.5)	3 (12.5)	
T-stage			.92
T0	1 (4.2)	0 (0)	
T1	5 (20.8)	6 (25.0)	
T2	2 (8.3)	2 (8.3)	
T3	9 (37.5)	11 (45.8)	
T4	7 (29.2)	5 (20.8)	
N-stage			.67
N0	5 (20.8)	3 (12.5)	
N1	9 (37.5)	12 (50.0)	
N2	9 (37.5)	7 (29.2)	
N3	1 (4.2)	2 (8.3)	
Stage (AJCC 8th)			.83
I	2 (8.3)	1 (4.2)	
II	3 (12.5)	4 (16.7)	
III	11 (45.8)	13 (54.2)	
IVA	8 (33.3)	6 (25.0)	
WHO Subtype			>.99
Type 1 (KSCC)	0	0	
Type 2a (NKDC)	0	1 (4.2)	
Type 2b (NKUC)	24 (100.0)	23 (95.8)	
Radiation Alone	2 (8.3)	1 (4.2)	>.99
Induction Chemotherapy	6 (25.0)	3 (12.5)	.46
Concurrent Chemotherapy	22 (91.7)	23 (95.8)	>.99
Type of concurrent chemotherapy			.38
High dose cisplatin	13 (59.1)	9 (39.1)	

© 2021 Li X et al. *JAMA Network Open.*

Weekly cisplatin	9 (40.9)	14 (60.9)	
Adjuvant Chemotherapy	11 (45.8)	14 (58.3)	.56
RT dose, median (IQR)	70.0 GyE (69.96-70.0)	69.96 Gy (69.96-69.96)	>.99

Abbreviations: IMPT, intensity-modulated proton therapy; IMRT, intensity modulated radiation therapy; RT, radiotherapy; IQR, interquartile range; KPS, Karnofsky Performance Score; AJCC, American Joint Committee on Cancer; WHO, World Health Organization; KSCC, keratinizing squamous cell carcinoma; NKDC, non-keratinizing differentiated carcinoma; NKUC, non-keratinizing undifferentiated carcinoma; GyE, Gray equivalent.

a. Categorical variables were assessed by Chi-squared test or Fisher's exact test. Continuous variables were compared by Wilcoxon rank sum test.

© 2021 Li X et al. *JAMA Network Open.*

eTable 5. Univariable and Multivariable Analyses for Progression-Free Survival in Propensity Score–Matched Cohort

	**Univariable**		**Multivariable[a]**	
**Variable**	**HR (95% CI)**	**P**	**HR (95% CI)**	**P**
RT Modality				
IMRT	Reference			
IMPT	0.31 (0.07-1.47)	.14	0.33 (0.07-1.68)	.18
Sex				
Female	Reference			
Male	3.55 (0.76-16.7)	.11	3.55 (0.72-17.5)	.12
Age	1.00 (0.96-1.05)	.97	0.98 (0.94-1.03)	.48
Smoking History				
No	Reference			
Yes	5.69 (1.43-22.7)	**.01**	6.33 (1.16-34.6)	**.03**
KPS	0.92 (0.80-1.05)	.20	1.00 (0.85-1.17)	.96
T-stage				
T3-T4	Reference			
T1-T2	0.45(0.12-1.69)	.24	0.36 (0.08-1.67)	.19
N-stage				
N2-N3	Reference			
N0-N1	0.59 (0.18-1.87)	.37		
Stage (AJCC 8th)				
III-IVA	Reference			
I-II	0.56 (0.12-2.58)	.46		
Concurrent Chemo				
No	Reference			
Yes	0.45 (0.06-3.63)	.45		
Type of Concurrent Chemo				
Weekly cisplatin	Reference			
High dose cisplatin	1.41 (0.43-4.58)	.57		

a. Multivariable Cox proportional hazard model included a panel of covariates determined *a priori*: radiotherapy modality, sex, age, smoking history, KPS and disease stage.

© 2021 Li X et al. *JAMA Network Open.*

eMethods. Details of Pretreatment Evaluation, Radiation Technique, Chemotherapy Regimen, and Follow-up Strategy

Pretreatment Workup

Pretreatment evaluation included history and physical examination, endoscopic evaluation of the nasopharynx, computed tomography (CT) and/or magnetic resonance imaging (MRI) of the nasopharynx including the neck, pre-treatment EBV status (plasma/tissue), baseline audiogram and dental evaluation, pre-chemotherapy laboratory tests including complete blood count, renal and hepatic function tests. Positron emission tomography (PET)/CT was done to rule out distant metastasis.

Treatment

Patients with stage I disease were treated with radiation alone. Stage II-IVA patients were treated with either concurrent chemoradiation followed by adjuvant chemotherapy or per protocol if patient is enrolled in NRG-HN001: Randomized Phase II and Phase III Studies of Individualized Treatment for Nasopharyngeal Carcinoma Based on Biomarker Epstein Barr Virus (EBV) Deoxyribonucleic Acid (DNA). In brief, after concurrent chemoradiation with cisplatin, patients on NRG-HN001 (ClinicalTrials.gov ID: NCT02135042) were reassessed using post-chemoradiation plasma EBV DNA to determine adjuvant therapy options. Induction chemotherapy was allowed in off-protocol patients.

Radiation therapy

Planning CT and MRI were acquired in the supine position with five-point thermoplastic mask immobilization and co-registered with baseline PET/CT. Gross tumor volume (GTV) was delineated based on imaging studies and clinical exam findings for both primary disease (GTVp) and involved lymph nodes (GTVn). Three-millimeter (mm) margin was added to the GTV to generate clinical target volume (CTV). High-risk CTVs including areas at risk of subclinical disease were generated as CTVp and CTVn. High-risk CTVp included the GTVp along with all the normal areas of spread, i.e skull base, parapharyngeal fat, posteriorly maxillary sinus. High-risk CTVn included GTVn and encompassed bilateral nodal level II-V and bilateral retropharyngeal nodal regions. Low-risk subclinical areas including level IV, VB and supraclavicular area without grossly involved low-lying nodes may be treated with the next lower dose level. Level IB was not included for node negative patients. Inclusion of IB level was at the discretion of the treating radiation oncologist for node positive patients. For IMRT planning, PTVs were generated from CTVs plus 3-5 mm margin to account for daily setup variation. For IMPT, CTV_eval was generated from CTVs plus 2-3 mm margin for optimization. The prescription doses were delivered to PTVs in IMRT and to CTV_eval in IMPT. The prescription doses to the gross disease/high-risk areas/low-risk subclinical areas were 69.96/56-59.4/54.12 GyE in 33 fractions or 70/59-63/56 GyE in 35 fractions. Radiation therapy was delivered once daily, five days per week. All target volumes were treated simultaneously in IMRT with integrated boost and sequentially in IMPT.

Radiation planning was done on Eclipse treatment planning system (Varian, Palo Alto, CA). Radiation therapy was delivered by linear accelerators (Varian, Palo Alto, CA) for IMRT and by the Proteus 235 system (Ion Beam Applications, Louvain-la-Neuve, Belgium) for IMPT. The Monte Carlo algorithm was used for plan calculation accounting for 3-5 mm setup variations and ±3.5% range uncertainties for robustness. A relative biological effectiveness (RBE) of 1.1 was used for IMPT optimization. IMPT planning was done using either single-field optimization or multi-field optimization. Proton beam orientation was selected to avoid potential heterogeneity within structures, for example, air in the nasal cavity and fluid in the sinuses. Typically, 3 beam directions of posterior, left anterior oblique and right anterior oblique fields were used for IMPT. All patients were aligned on a 6 degree-of-freedom treatment couch and underwent daily image-guided radiation therapy.

Chemotherapy

Concurrent chemotherapy was administered with cisplatin, either weekly (40mg/m^2) up to 7 cycles or every three weeks (100mg/m^2) up to 3 cycles. Induction chemotherapy regimen was administered at the discretion of treating medical oncologist and included cisplatin, carboplatin, gemcitabine, paclitaxel, 5-fluorouracil (5-FU). Adjuvant chemotherapy for patients on NRG-HN001 included cisplatin/5-FU or gemcitabine/paclitaxel. Adjuvant chemotherapy for off-protocol patients was administered at the discretion of treating medical oncologist.

Follow up

All patients were seen by treating radiation oncologist weekly for toxicity evaluation during treatment. Patients were subsequently followed at 8-12 weeks post radiation, then at 3-month interval for the first 2 years, then every 6-12 months thereafter. Follow up imaging included MRI of the nasopharynx and the neck, and PET/CT at appropriate intervals determined by the treating physician. Fiberoptic nasolaryngoscopy examination, focused head and neck physical examination, and toxicity evaluation were done at every radiation oncology follow up visit.

© 2021 Li X et al. *JAMA Network Open.*
